# Pharmacologic prophylaxis for atrial fibrillation following cardiac surgery: a systematic review

**DOI:** 10.1186/1749-8090-5-121

**Published:** 2010-11-30

**Authors:** Ioanna Koniari, Efstratios Apostolakis, Christina Rogkakou, Nikolaos G Baikoussis, Dimitrios Dougenis

**Affiliations:** 1 Cardiothoracic Surgery Department. Patras University, School of Medicine. Rion Patras, Greece

## Abstract

Atrial Fibrillation (AF) is the most common arrhythmia occurring after cardiac surgery. Its incidence varies depending on type of surgery. Postoperative AF may cause hemodynamic deterioration, predispose to stroke and increase mortality. Effective treatment for prophylaxis of postoperative AF is vital as reduces hospitalization and overall morbidity. Beta - blockers, have been proved to prevent effectively atrial fibrillation following cardiac surgery and should be routinely used if there are no contraindications. Sotalol may be more effective than standard b-blockers for the prevention of AF without causing an excess of side effects. Amiodarone is useful when beta-blocker therapy is not possible or as additional prophylaxis in high risk patients. Other agents such as magnesium, calcium channels blocker or non-antiarrhythmic drugs as glycose-insulin - potassium, non-steroidal anti-inflammatory drugs, corticosteroids, N-acetylcysteine and statins have been studied as alternative treatment for postoperative AF prophylaxis.

## Introduction

Atrial Fibrillation (AF) is the most common arrhythmia occurring after cardiac surgery and its peak incidence is between second or third postoperative day. Postoperative AF ranges depending on surgery type. Especially, AF occurs in nearly 30% of patients undergoing coronary bypass grafting (CABG), and in 40% and 50% of patients after valve surgery alone or combined valve and CABG surgery respectively [1,2]. Pathophysiologic parameters such as the abnormal electrophysiological state of the atria, the unequal shortening of the atrial myocytes refractory period as well as variable conduction speed through the atrial tissue predispose to the development of AF. It is also considered that ischemia of the atrial tissue, increased sympathetic activation, and exaggerated inflammatory response may play a triggering role in the development of postoperative AF [3]. Risk factors of postsurgical AF could be divided into: preoperative, intra-operative and postoperative. Preoperative factors mainly include: a. atrial tissue damages due to age, previous rheumatic fever, elevated left ventricular diastolic pressure, hypertension and coronary syndromes [4-9], b. heart diseases such as left ventricular hypertrophy, left atrium enlargement or history of congestive heart failure [4,10], and c.electrolytic imbalance such as hypokalemia, hypomagnesemia, hypothyroidism, preoperative use of digoxin or milrinone [4,11]. Finally, obesity, male gender, chronic obstructive pulmonary disease (COPD), tachycardia, prolonged P-wave deviation may also predispose to AF [10,12-19]. While, intra-operative risk factors could be attributed to increased sympathetic activation due to stimulation of catecholamines, reflex sympathetic activation from volume loss, anemia, pain, adrenergic drug administration, aortic cross clumping duration, early return of atrial electrical activity after cardioplegia, bicaval venous cannulation, left ventricular venting via pulmonary vein as well as extracorporeal circulation [4,6,14,18]. Postoperative AF may be correlated with hemodynamic deterioration (myocardial infarction, heart failure, thromboembolism, bleeding due to anticoagulation), stroke, hypomagnesemia [15], extubation time [16], and others as increase in postoperative P-wave dispersion [17] and exaggerated inflammation reaction [18-21]. Consequently, the effective treatment for the prevention of postoperative AF is of vital importance. Numerous pharmacologic strategies attempt to reduce the incidence of postoperative AF. Overall, most reported studies demonstrate a positive effect with a variety of pharmacologic agents either anti-arrhythmic (β-blocker, amiodarone, magnesium, calcium blocker) or non-antiarrhythmic drugs (glycose-insulin-potassium, non-steroidal anti-inflammatory drugs, corticosteroids, N-acetylcysteine, statins); to date, however, no signal particular agent or combination of agent have completely eliminated post cardiac surgery AF.

## Pharmacologic prophylaxis for postoperative atrial fibrillation

### Beta-blockers

All identified meta-analyses demonstrated that b-blockers significantly reduced the incidence of postoperative AF [9-13]. Particularly, Andrews et al, showed that the incidence of post-CABG AF decreased from 34% to 8, 7% in patients received b-blockers. In another meta-analysis of Kowey et al, the decrease in incidence of AF was from 20% to 8, 7% [22]. However, Crystal et al performed the largest meta-analysis based on 27 randomised controlled trials that included 3.840 patients. Especially, the control group presented an incidence of AF approximately 33%, while notably patients receiving b-blockers had an incidence of 19% [23]. Ferguson et al [24], in another large retrospective analysis of the Thoracic Surgeons surgical database that included 629.877 patients, observed the morbidity and mortality rate associated with the peri-operative use of b-blockers. Notably, they revealed a reduction in mortality rate from 3.4% to 2.8% in patients that received peri-operatively b-blockers. Numerous randomized trials have been conducted so as to evaluate the effectiveness of b-blockers in the prevention of AF. In b-Blocker Length of Stay (BLOS) trial, Connolly et al, evaluated the efficacy of metoprolol against placebo therapy in preventing postoperative AF in 1000 patients undergoing cardiac surgery [25]. In all, 85% of patients had CABG surgery and the remainder had valve surgery or combined valve and CABG surgery. The administered daily dose of metoprolol was 100 or 150 mg starting immediately after the surgery and continued until discharge from the hospital. The incidence of postoperative AF was significantly lower in metoprolol group (31%) compared to placebo (39%), representing a relative risk reduction of 20%.

Similarly, Lucio et al, randomized 200 patients underwent isolated CABG to receive either metoprolol or no drug [26]. Metoprolol was given orally adjusted to maintain optimal heart rate and started from the 12th hour to the 7th postoperative day or hospital discharge. Postoperative AF and flutter occurred at 24% in control versus 11% in metoprolol group (p = 0.02). Tsuboi et al, randomized 160 patients who underwent scheduled isolated CABG to receive either carvedilol or not [27]. Postoperative paroxysmal AF was 15% in carvedilol group (p = 0.009). White et al, assigned randomly 41 patients after CABG to receive prophylactic timolol or placebo. Timolol 0.5 mg diluted in 10 ml of saline was given IV over 1 min twice daily only when patient condition was stable, following oral timolol twice daily for 7 days. Timolol decreased significantly (p < 0.05) the episodes of supraventricular tachycardia as well as of AF and/or flutter [28]. Lamb et al, randomized 60 patients underwent CABG treated with atenolol or not. Remarkably, 37% of patients in control group experienced a sypraventricular arrhythmia compared to 3% in atenolol group (p = 0.001) [29]. Several studies compared the efficacy of iv or oral b-blocker as well as different types of b-blocker. Halonen et al, in an attempt to compare the iv with the oral use of metoprolol, randomized 240 patients who underwent first on pump CABG, aortic valve replacement or combined aortic valve replacement and CABG [30]. In both groups, the metoprolol administration was based on heart rate for a 48 hour period. Postoperative AF presented a significant decrease (p = 0.036) in IV group (16.8%) compared to oral group (28.1%). It should be mentioned that patients at risk to develop complications associated with IV metoprolol were excluded. Also, Maniar et al randomized 47 patients for elective CABG to receive either esmolol IV or standard oral β-blocker (propanolol/metoprolol) [31]. Esmolol was given within 6 to 18 hours of arrival to recovery room, continued for up to 24 hours and then these patients transitioned to oral β-blocker. The incidence of postoperative AF was the same (26%) in both groups. However, patients in esmolol group developed significant adverse effects (hypotension, symptomatic bradycardia, CHF) compared to oral β-blocker. An important limitation was that patients in oral β-blocker group received greater number of bypass grafts than in the esmolol group. Similarly, Balcetyte - Harris et al [32], showed that the tolerance to esmolol was poor, and that its effectiveness in the prevention of atrial fibrillation was not better than oral b-blockers.

Moreover, comparison of the effectiveness between metoprolol and carvedilol has also been performed. Especially, Acikel et al [33], randomized 110 patients scheduled for elective CABG to receive either metoprolol (50 mg td) or carvedilol (12, 5 mg td). Therapy was started 3 days prior to surgery and continued in the postoperative period with mean dosages of carvedilol (13 mg daily) and metorpolol (58 mg/day) in corresponding groups. Postoperative AF had an incidence of 36.4% in metorpolol compared to 16% in carvedilol group (p = 0.029). Also, Hafgjoo et al [34], randomized 120 patients underwent CABG to receive metoprolol or carvedilol. In this study, the therapy was started 10 days prior the surgery and initiated with an oral dose of carvedilol 6.25 mg and 25 mg metoprolol twice daily respectively. Then the dosage was increased until the maximum tolerated dose. The incidence of postoperative AF was significantly reduced (p = 0.022) in carvedilol (15%) compared with metoprolol (33%) group. However, the study presented several limitations: it was a single centre study consisted of small number of patients and thus, inflammation markers such as CRP had not been measured, despite the hypothesis that anti-inflammatory properties of carvedilol may have contributed to increased efficacy. Finally, several studies compared atenolol with other regiments such as digitalis or propafenone. Yazicioglu et al [35], randomized 160 patients underwent CABG into 4 groups of treatment: a. digoxin and atenolol, b. digoxin, c. atenolol, d. placebo. The combination of atenolol and digitalis (5%) decreased significantly postoperative AF compared with placebo (25%, p = 0.012) but there was no significant difference compared to digoxin or atenolol alone (17.9%, 15.4% p = 0.087). Merrick et al [36], in SPPAF trial allocated randomly 207 patients underwent non-emergency cardiac surgery, to receive orally either propafenone 300 mg twice daily or atenolol 50 mg once daily from the first until the 7th postoperative day or until an end point (AF appearance) was reached. The atenolol and the propafenone presented equal efficacy (10, 7% vs. 12%) in the prevention of AF.

### Sotalol

Sotalol is a b-blocker that also disposes Class III anti-arrhythmic characteristics. The effectiveness of sotalol has been proved in placebo control trials [37,38]. Pfisterer et al [37], randomized 255 patients, who referred for CABG or aortic valve operation, to receive either 80 mg of sotalol orally or matching placebo. Sotalol reduced significantly (p = 0.0012) the rate of postoperative sypraventricular tachy-arrhythmia from 46% (placebo) to 26% as well as the length of hospital stay (p < 0.05). Preoperative β-blockers therapy was stopped before the surgery, fact that might have been responsible for increasing incidence of SVA arrhythmia in placebo group. Gomes et al, randomized 130 patients underwent open heart surgery, to receive sotalol (80 mg to 120 mg) or placebo [38]. Sotalol significantly decreased (p < 0.001) postoperative AF (12.5%) compared to placebo (38%). An important limitation was the low number of participants. Several randomized controlled trials compared sotalol to conventional b-blockers. The largest study was by Suttorp et al [39,40], who performed a four-arm study comparing low or high doses of sotalol or propranolol in 429 patients. Sotalol 40 mg tds resulted in an incidence of 14% of AF compared with 19% incidence of low dose propranolol, revealing no statistical significance. Auer et al [41] randomized 312 patients underwent cardiac surgery into four groups: 1. metoprolol in combination with oral amiodarone, 2. metoprolol, 3. Sotalol, 4. placebo. The incidence of AF was 32% with sotalol and 40% with metoprolol, although this was again non-significant. Combined metoprolol and amiodarone as well as sotalol had a significant lower frequence of AF than placebo (30.2%, 31.7%, 53.8% respectively P < 0.01). Sanjuan et al [42] studied 253 patients and demonstrated a significant reduction of AF from 22% to 10% comparing atenolol with sotalol. Janssen et al [43], randomized 130 patients to sotalol, metoprolol or no therapy. Only 2.4% of patients receiving sotalol went into AF, compared with 15% in the metoprolol group and 36% of controls, which was a significant finding. Parikka et al [44] randomised 191 patients to receive either sotalol or metoprolol. Postoperative AF observed in only 16% of patients receiving sotalol compared with 32% of those receiving metoprolol (p < 0.01). Nystrom et al [45], randomised 101 patients to high dose sotalol or (1/2) dose b-blockers. Postoperative AF occurred in 10% of patients in sotalol group compared with 29% in the b-blocker group, revealing a statistical significant diferrence (p = 0.028). Abdulrahman et al [46], randomized 191 patients to sotalol or metoprolol. The incidence of AF was 10% in the sotalol group and 22% in the metoprolol group. Finally, Crystal et al [47], briefly summarized these studies and demonstrated that the incidence of AF in the sotalol groups was 12% compared with 22% in the b-blocker groups, which was a significant finding. In these studies, either 40 mg tds or 80 mg bd were safe but doses higher than those associated with a higher incidence of side effects. Wunderman et al [48], performed a meta-analysis including 10 randomized trials (1403 patients) comparing sotalol and amiodarone. Incidences of postoperative AF in sotalol group were 21.5% versus 14.1% in amiodarone group, presenting no significant difference. Also, the adverse effects that required drug discontinuation as well as the length of hospital stay was similar between two regiments. Sotalol can be proarrhythmic as in non-surgical patients the proarrhythmic risk has been reported to be 4.3-5.9%. Because of the proarrhythmic effects of sotalol, ordinary beta-blockers are a safer alternative to sotalol in the prevention of AF after surgery [2].

### Amiodarone

Amiodarone has been proved to be useful in the prevention of postoperative AF. Mitchel et al, in the PAPABEAR trial randomized 600 patients, who were listed for non-emergent CABG and/or valve replacement/repair surgery, to receive amiodarone or placebo [49]. In amiodarone group (n = 299) amiodarone was given orally 10 mg/kg/day 6 days prior to surgery through 6 days after the surgery (13 days), whereas placebo was administered for the same period. Remarkably, amiodarone reduced significantly (p < 0.001) postoperative AF incidence (16.1%) compared to placebo (29.5%). Also, Daud et al [50], randomized 124 patients underwent elective cardiac surgery to receive oral amiodarone (600 mg/day prior and 200 mg/day after surgery) or placebo for 7 days prior the surgery until the discharge. Similarly, amiodarone presented a statistically significant (p = 0.03) decrease in postoperative AF incidence (23%) compared to placebo (42%). Redle et al, evaluated 150 patients undergoing CABG in a randomized double blind controlled trial, comparing amiodarone with placebo [51]. In amiodarone group, the two grams were given in a graduated dosing schedule and then the patients received 400 mg/day beginning on the first postoperative day and continued for seven days. The incidence of postoperative AF was not affected by the prophylactic oral amiodarone as there was no difference between the two groups (p = 0.3). A serious study limitation was that the contaminant use of digoxin, calcium channel blocker and β-blocker was not controlled.

The duration and dosage of amiodarone has also been evaluated. Especially, Giri et al, in AFIST I trial divided randomly 220 patients over 60 years of age into two groups: amiodarone and placebo [52]. Amiodarone was given orally beginning either the first postoperative day at dosage of 6 gr over 6 days or the 5th preoperative day at dosage of 7 gr over 10 days. The incidence of postoperative AF was reduced in amiodarone (28%) group compared to placebo (38%) but without revealing any significant difference (p = 0.01). White et al [53], in AFIST II, trial randomized 160 patients underwent cardiothoracic surgery to amiodarone or placebo and then to pacing or no pacing using a 2×2 fractional design. All therapies began within 6 hrs post surgery. Amiodarone was given by intravenous infusion for the first 24 hrs (1050 mg total) followed by oral therapy (400 mg three times daily) for 4 postoperative days (4800 mg total). Atrial septal pacing was given for 96 hrs. Amiodarone reduced the risk of AF by 43% and the risk of symptomatic AF by 68% (p = 0.037 and p = 0.019) versus placebo. Atrial septal pacing did not reduce AF or symptomatic AF incidence compared to no pacing. Notably, the risk of postoperative AF in patients receiving amiodarone and pacing was lower than the placebo/no pacing and the placebo/pacing groups (57.9% and 60.5% reductions, p = 0.047 and p = 0.040 respectively).

The effect of intravenous amiodarone therapy has also been investigated. Guarnieri et al [54], in ARCH trial randomized 300 patients underwent open heart surgery to amiodarone infusion or placebo. The drug infusion was started within 3 hours of entering the surgical ICU amiodarone was infused at rate of 1 gr over 24 hrs for 2 days (2 g total). Postoperative atrial fibrillation occurred 35% in amiodarone group versus 47% in placebo, revealing no statistically significant difference (p = 0.01).

Similarly, Yagdi et al [55], randomized 157 patients to amiodarone infusion or placebo. Amiodarone infusion without a loading dose was given at a dose of 10 mg/kg/day within 2 hours of entering the cardiovascular ICU for 48 hours. On 2nd postoperative oral amiodarone was initiated at 600 mg/day three times daily for 5 days, 400 mg per day twice daily for the following 5 days, and 200 mg per day in a single dose for the last 20 days. Amiodarone did not reduce significantly postoperative AF incidence compared to placebo (19, 4% vs 25%). Kerstein et al [56], randomized 143 patients that were scheduled for CABG to amiodarone infusion or placebo. IV amiodarone, 0.73 mg/min, without any loading dose was administered on call to the operating room for 48 h, and followed by oral amiodarone, 400 mg q12 h, for the next 3 days. Atrial fibrillation occurred in 3 of 51 patients (5.88%) in the amiodarone group, compared to 24 of 92 patients (26.08%) in the control group, presenting no statistical significant difference. Of note, most patients also received b-blockers and this study is limited by its non-randomised design. Also, Lee et al [57], began i.v. amiodarone 3 days before CABG and continued it for 5 days after surgery. The incidence of AF was lower and the duration shorter in the amiodarone group than in the placebo group (12% vs. 34%), respectively. Doerge et al [58], randomized 150 patients into amiodarone or placebo groups. Amiodarone given IV for 3 days following surgery did not decrease the incidence of AF. Treggiari-Venzi et al [59], conducted a randomized controlled double-blind trial in which patients received amiodarone postoperatively (900 g/days for 72 h) and demonstrated that the decrease in AF was not statistically significant.

The efficacy of amiodarone has also been compared with other agents such as b-blockers, sotalol, digoxin and diltiazem. Especially, Tokmakoglu et al [60], allocated randomly 241 patients, undergoing elective CABG into three groups. Patients in first group (i) received metoprolol 100 mg/24 h per oral preoperatively, 2×0.5 mg digoxin intravenously in the early postoperative period and 0.25 mg digoxin in combination with 100 mg metoprolol per os on the first postoperative day until discharge. Patients in second group (ii) received totally 1200 mg IV/24 hrs amiodarone which the 300 mg- bolus dose/1 hour was given as soon as the operation had been finished. On the next day patients were given 450 mg/24 h amiodarone IV and then 600 mg/day in three doses per os until discharge. Third group was the control group with no prophylaxis. AF occurred in 16.8%, 8, 3% and 33.6% of patients in group i, ii and iii respectively. Both study groups were significantly effective in the prevention of post-CABG AF with respect to control group (p < 0.01 in group i and p < 0.001 in group ii versus control). Sleilaty et al [61], randomized 200, admitted for elective CABG to receive oral amiodarone or oral bisoprolol beggining 6 hrs after surgery. Amiodarone patients received 15 mg/kg, followed by 7 mg/kg/d until discharge and then 200 mg/d for one month. The patients in bisoprolol group received 2.5 mg then 2.5 mg bid bisoprolol indefinitely in the postoperative period. Postoperative AF occurred in 15.3% of the patients in the amiodarone group and 12.7% of the patients in the bisoprolol group showing no significant difference concerning the onset time of AF episode, total duration and recurrence of AF. On the contrary, Solomon et al [62] performed a randomized study on 102 consecutive patients undergoing cardiovascular surgery. The patients were randomized to receive amiodarone (1 gr/day intravenously for 48 hrs, then 400 mg/day orally until discharge) or propranolol (1 mg intravenously every 6 hrs for 48 hrs, then 20 mg orally four times a day until discharge). The incidence of postoperative AF was significantly lower in amiodarone group (16%) compared to propranolol treated patients (32.7%, p = 0.05), showing the superiority of amiodarone. Mooss et al, in REDUCE trial evaluated 160 patients underwent CABG, combined CABG and AVR surgery, or AVR surgery alone [63]. Patients were randomized to receive either sotalol 80 mg twice daily or intravenous amiodarone 15 mg/kg over 24 hrs followed by oral amiodarone 200 mg three times daily. Postoperative AF occurred in 17% of patients randomized to amiodarone and in 25% of those randomized to sotalol, revealing no significant difference (p = 0.21) and further similar efficacy between two regiments. On the other side, Mikroulis et al, randomized 120 patients underwent CABG to receive amiodarone (300 mg IV, followed by 1 gr IV daily for 48 hrs, then 400 mg IV daily for further 48 hrs) or diltiazem(continuous infusion/minimum dose 0.1 mg/kg/h) followed by an oral b-blocker for the remainder of their hospitalization [64]. The incidence of post-CABG AF was not significantly different between amiodarone (11.7%) and diltiazem (10%).

Finally, meta-analyses concerning prophylactic effect of amiodarone in prevention of postoperative cardiac surgery have also been performed. Particularly, Bagshaw et al, performed a meta-analysis included 19 randomized control trials (3295 patients) of amiodarone [65]. Amiodarone significantly reduced the odds ratio of AF (p < 0.0001), ventricular tachyarrhythmias (p < 0.0001), strokes (p = 0.02) as well as duration of hospitalization (p < 0.0001). Also, Haan et al [66], evaluated 7 randomized control trials including 1064 patients. They concluded that amiodarone decreased the incidence of postoperative AF in all of the studies, and reached statistical significance in two [57,66]. Patel A et al [67], analyzed 18 randomized controlled trials enrolling 3408 patients so as to assess the safety of amiodarone in prevention of postoperative AF. Notably, they showed that amiodarone is associated with an increased risk of developing bradycardia and hypotension especially when average daily dose of IV amiodarone exceeds 1 gr. Finally, Crystal et al [47], summarized ten randomized controlled trials and reported an incidence of AF of 22.5% in the amiodarone groups and an incidence of 37% in control groups.

### Magnesium

Low magnesium concentrations are independent risk factors of AF after cardiovascular surgery. Several studies have been conducted using magnesium as prophylaxis agent postoperatively.

Kohno et al, evaluated 200 patients who underwent isolated initial CABG operation in a not randomized retrospective study [68]. The first 100 patient did not receive prophylactic treatment, whereas the next 100 patients were treated with 10 mmol of magnesium sulfate infused IV daily for 3 days after surgery. The incidence of post-operative atrial fibrillation was 35% in the untreated group compared with 16% in the magnesium group (p = 0.002). An important limitation is the lack of randomization and the nature of retrospective analysis that have weakened the cogency of the study.

Kaplan et al [69], conducted a randomized study on 200 consecutive patients in whom they performed initial elective CABG. In treatment group 100 patients received 3 g magnesium intravenously preoperatively, perioperatively and for the 3 following postoperative days. No significant difference was found compared to the control group, although in a sub-analysis of patients who had low pre-operative serum magnesium, a significant reduction (p < 0.05) in AF was demonstrated. Yeatman et al [70], performed the largest study on magnesium prophylaxis. Especially, 400 patients were randomized in a double blind fashion to receive 40 mmol of 2 mmol/ml magnesium sulphate in the cardioplegia solution or controls. The incidence of AF was 22% in the magnesium group compared with 29% in controls, which was non-significant, although the findings were significant in a subset analysis of urgent patients. However, authors acknowledged that they should have used a higher dose of magnesium to obtain a concentration nearer to 15 mmol/l of cardioplegia, as their dose only produced a concentration of 5 mmol/l. Similarly, Zangrillo et al [71], randomized 160 consecutive patients underwent elective isolated, off-pump CABG to receive either 2.5 gr magnesium sulphate infusion intraoperatevely over 30 minutes or normal saline solution. Postoperative atrial fibrillation occurred in 20% of patients treated with magnesium and in 22.5% of patients in placebo group (p = 0.9), revealing no statistical difference between the two groups. On the contrary, Toraman et al, performed an randomized contolled study in 200 patients, giving them either 6 mmol of magnesium both pre-operatively and post-operatively or placebo [72]. Only 2% of patients receiving magnesium went into AF compared with 21% in the control group. Unfortunately, patients receiving b-blockers or digoxin were excluded. Also, Hazelrigg et al, randomized 105 patients to receive 80 mg/kg of magnesium pre-operatively, then 8 mg/(kg h) post-operatively for 48 h or placebo in 97 patients [73]. Thirty two patients treated with magnesium went into AF compared with 41 control patients, which was a non-significant trend towards benefit. However, the reduction in AF was significantly different between groups on day 1. Fanning et al, performed a randomized study in which patients received either magnesium 178 mEq or placebo for 4 days following surgery, showing that the incidence of AF was lower in the magnesium group [74]. Moreover, Maslow et al, conducted a retrospective study included patients undergoing beating heart CABG and demonstrated that magnesium treated patients were less likely to experience postoperative AF than other patients (12% vs. 29%) [75]. On the other hand, Wistbacka et al [76], performed a double-blind study so as to assess the dosage of magnesium in the prevention of AF. Of note, the highest dose of magnesium (4.2 g before surgery, 11.9 g infusion the first postoperative day and 5.5 g the following day) decreased the incidence of AF more than lower doses (4.2 g, 2.9 g, 1.4 g). Jensen et al instead found that magnesium decreased the duration of AF and flutter, but did not decrease the incidence of AF [77].

In the meantime, there are also negative studies about the preventive effect of magnesium. Particularly, Parikka et al, performed a study in which 70 mmol of magnesium was given in the first 48 h after surgery [78]. No effect on the incidence of AF was seen, while a high serum magnesium level increased the incidence of AF. In a study of Karmy-Jones et al [79], 14.4 g of magnesium was given during the first 24 h postoperatively but no effect of magnesium on the incidence of supraventricular tachycardia was shown.

Magnesium has also combined or compared with other agents such as sotalol. Aerra et al, evaluated 103 consecutive coronary patients that received sotalol and magnesium or placebo [80]. These patients received 40 mg of sotalol orally twice daily from the first post-operative day for 6 weeks and 2 g of magnesium intravenously immediately post surgery and on the first post-operative day. The incidence of atrial fibrillation in the sotalol and magnesium group was 13.5% compared to 27.0% in the controls (p = 0.025). However, the study had serious limitations: retrospective, not randomized and under one surgeon's care. In addition, Forlani et al [81], performed a randomized controlled trial, separating 207 patients into four groups. Patients received either sotalol 80 mg bd or magnesium 1.5 g orally for 6 days postoperatively or both or neither treatment. Remarkably, only 1 of 52 patients who received both treatments went into AF compared with 19 of 50 control patients. In contrast, Bert et al [82], performed a multi-arm study in 387 patients randomized into six groups of prophylaxis: control, magnesium only, digoxin only, magnesium and digoxin, propranolol only, and magnesium and propranolol. Patients randomized to a regimen including magnesium received 12 g given during 96 hours postoperatively. Unfortunately, addition of magnesium had no beneficial effect as compared with b-blockers, digoxin or controls.

Several meta-analyses concerning magnesium have also been published. Shiga et al [83], performed a meta-analysis included 17 randomized control trials (2069 patients) summarising papers that contained magnesium alone as prophylaxis and compared it to placebo treatment. Magnesium supplementation reduced significantly the risk of supraventricular arrhythmias (p = 0.002) after cardiac surgery by 23%, of AF by 29% and of ventricular arrhythmias by 48% (p < 0.0001). However, magnesium had no notable effect on length of hospitalization, incidence of myocardial infarction or mortality. They also summarised the complications reported in 648 patients. They found no episodes of bradycardia or hypotension. Of note, important differences were found between all these studies and no one prophylactic regime was found to be superior to another. Regimes ranged from a single dose of 5 mmol in the cardioplegia solution to 110 mmol over the course of 3 days. Miller et al, performed a meta-analysis included 20 randomized trials with 2490 patients [84]. They showed that postoperative AF was reduced from 28% in the control group to 18% in the treatment group with significant heterogeneity between the trials. Also, magnesium did not significantly reduce hospitalisation duration or mortality. Again, they did not recommend one specific magnesium prophylactic regimen. Finally, the most recent meta-analysis by Alghamdi et al [85], summarized only eight randomized controlled trials that compared magnesium with placebo. They also found a highly significant reduction in relative risk with the addition of magnesium.

## Other pharmacological prevention

Nonsteroidal anti-inflammatory medications have also been tested as prophylaxis of post-CABG AF. Cheruku et al [86], randomized 100 patients to receive either ketorolac 30 mg IV/6 hour followed by ibuprophen 600 mg p.o. three times daily for 7 days or no drugs. Of note, postoperative AF was reduced from 28.6% (control group) to 9.8% in the ibuprophen group (p = 0.017). Two randomized controlled trials have also been conducted concerning the effect of corticosteroids. Especially, Halonen et al [87], evaluated 241 consecutive patients, underwent first CABG and/or aortic valve replacement in a double-blind multicenter trial. Patients were randomized to receive either 100 mg hydrocortisone or placebo. The incidence of postoperative AF was significantly lower in hydrocortisone group (30%) compared to placebo (48%, p = 0.004). Also, Prasongsukarn et al [88], randomized 86 patients underwent elective first time CABG to 1 gr of methylprednisolone IV before surgery and 4 mg of dexamethasone IV every 6 hours for 1 day after surgery or placebo. Postoperative AF was significantly lower in steroid group (21%) compared to placebo group (51%, p = 0.003). However, patients in steroid group presented more complications and further prolonged hospitalization. Also, the effect of antioxidant agent N-acetylcysteine (NAC) has been evaluated in postoperative AF. Ozaydin et al [89], conducted a prospective; double-blind trial consisted of 115 patients undergoing CABG and/or valve surgery that randomized to NAC or placebo. The incidence of postoperative AF was lower in NAC (5.2%) compared to placebo (21.1%, p = 0.019) but the mean postoperative hospital stay was similar in both groups (p = 0.82). Bothe et al [90], evaluated 11 randomized trials (468 patients) referring to the effect of glucose-insulin-potassium therapy (GIK) after cardiac surgery. Particularly, the findings indicate that GIK may considerably improve postoperative recovery of contractile function and further reduce the incidence of postoperative AF. Finally, ARMYDA-3 a randomized, prospective, double-blind, placebo-controlled trial evaluated the effect of atorvastatin in reducing postoperative AF in 200 patients undergoing elective cardiac surgery [91]. Treatment with atorvastatin 40 mg/day initiated one week before surgery, significantly reduced the incidence of postoperative AF versus placebo (35% vs 57%, p = 0.003). On the contrary, Virani et al [92], conducted a retrospective cohort analysis consisted of 4044 patients underwent cardiac surgery without a history of chronic or paroxysmal AF that dived into two groups: those who received preoperative statin therapy and those who did not. They demonstrated that preoperative statin therapy was not associated with decreased incidence of postoperative AF including patients with severe left ventricular dysfunction.

## Conclusions

In conclusion, b-blockers should routinely be used as first choice for the prophylaxis of AF in all patients undergoing cardiac surgery, unless otherwise contraindicated (Grade A recommendation based on level 1a studies) [3]. Sotalol may be more effective than standard b-blockers for the prevention of AF without causing an excess of side effects (Grade A recommendation based on level 1b studies). Amiodarone should be used for prophylaxis of AF in all patients undergoing cardiac surgery in which b-blocker therapy is not possible (Grade A recommendation based on level 1a and1b studies). In high-risk patients receiving b-blocker therapy for prophylaxis of AF, amiodarone may also be used as additional prophylaxis with an acceptably low incidence of complications [3]. These patients should be protected from the complications of bradycardia with temporary pacing wires being placed intra-operatively (Grade A recommendation based on level 1b studies) [3].

## Competing interests

The authors declare that they have no competing interests.

## Authors' contributions

All authors: 1. have made substantial contributions to conception and design, or acquisition of data, or analysis and interpretation of data; 2. have been involved in drafting the manuscript or revisiting it critically for important intellectual content; 3. have given final approval of the version to be published.
